# Preparation of altrenogest soft capsules and their bioequivalence in gilts

**DOI:** 10.3389/fvets.2024.1468615

**Published:** 2024-08-30

**Authors:** Jinxia Xu, Lifang Ling, Yanhua Lan, Ye Yuan, Long Ran, Jiaxin Jiang, Xianhui Huang

**Affiliations:** Guangdong Key Laboratory for Veterinary Drug Development and Safety Evaluation, College of Veterinary Medicine, South China Agricultural University, Guangzhou, China

**Keywords:** altrenogest, softgel capsules, gilts, stability studies, bioequivalence study

## Abstract

**Introduction:**

Altrenogest (ALT) is widely used to regulate the estrous cycle of sows and mares; however, currently used oral solutions of ALT are deficient in terms of dose accuracy and stability during use. To resolve these problems, we aimed to prepare softgel capsules of ALT with a unit dose equal to the clinically administered dose.

**Method:**

The shell of the softgel capsule was mainly composed of gelatin and glycerol, with titanium dioxide and red iron oxide as masking agents. Using the shake flask method, the contents were screened for ethyl acetate as a lipophilic solubilizing carrier based on soya bean oil. The contents were automatically filled and compressed into softgel capsules by a softgel capsule machine. The quality of the prepared softgel capsules was evaluated through a drug content test, an *in vitro* release test, and stability studies. Furthermore, bioequivalence studies were conducted with atrenogest oral solution.

**Results:**

The content is an ALT concentration of 2% oil solution and the specification of the softgel is 20 mg/capsule. In *in vitro* dissolution experiments, the softgel capsules were rapidly disintegrated and released in three different pH buffers, with a cumulative release rate of nearly 100% at 1 h. The softgel capsules were stable at high temperature and under strong light for 10 days, and the concentration of ALT was >99% in the 6-month accelerated and long-term tests. In the bioequivalence study, Tmax of the softgel capsules was 2.20 ± 0.77 h, *t*_1/2_ was 6.36 ± 1.74 h, and *C*_max_ was 64.65 ± 20.69 ng/ml. The main pharmacokinetic parameters *T*_max_, *C*_max_, *AUC*__0−_t_, and *AUC*_0−∞_, did not differ significantly between the softgel capsules and the commercially available ALT oral solution (*P* > 0.05), and bioequivalence was demonstrated within the 90% confidence interval.

**Conclusion:**

The prepared softgel capsules have the advantages of higher content, ease of use with accurate dosing, good stability, and equivalence to ALT oral solution, implying that our softgels are ready for clinical use.

## Graphical Abstract

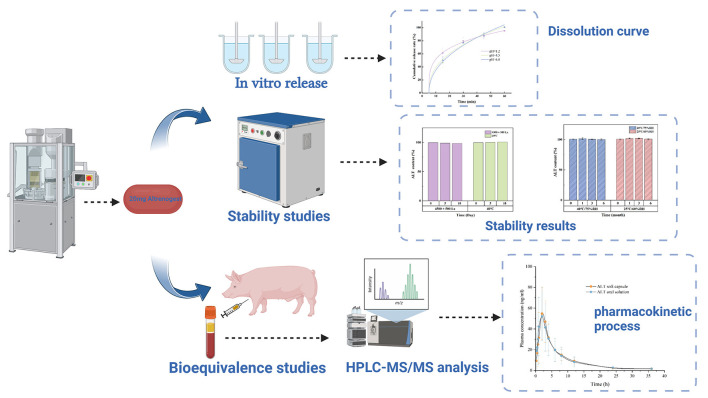

## 1 Introduction

The batch production model has become the trend in large-scale pig farming. It has the advantages of improving sow productivity, reducing biosecurity risks on pig farms, and improving the economic efficiency of farms (1, 2). The regulation of the estrous cycle of sows by exogenous hormones represents a significant method of enhancing herd homogeneity and achieving batch production (3). Hormonal drugs, including gonadotropin-releasing hormone (4), progesterone (5), and altrenogest (6) have the capacity to regulate the estrous cycle in sows. Nevertheless, altrenogest is currently the sole commercially available progestin for use in pigs that can be administered orally. Allylgestrin is extensively employed in the predominant swine farming regions of the globe, and its efficacy in regulating the estrous cycle of sows has been extensively documented (7, 8). Furthermore, altrenogest is not only straightforward to administer but also less irritating to sows than other hormone-based injectable drugs. Altrenogest (ALT) is a progesterone analog that exerts estrus suppression by inhibiting plasma secretion of the endogenous gonadotropins luteinizing hormone and follicle-stimulating hormone. When ALT is discontinued, plasma concentrations of these hormones gradually increase, and follicle growth and maturation are induced, resulting in a simultaneous estrous effect in the animals (9). In addition, many studies have shown that ALT treatment promotes uterine development (10), and improves reproductive performance (9, 11), farrowing rate, and newborn weight of reserve and primiparous sows (12).

Currently, the main clinically used dosage form is 0.4% ALT oral solution, initially developed by Intervet International Ltd. and administered at a dose of 5 ml/pig (20 mg/pig in terms of ALT) (13), which can be sprayed on the feed or fed directly into the sows' mouths with a dosing gun. However, ALT is a photosensitive drug, and if it is not consumed in time after spraying on feed, it is easily degraded by light (14). Also, uneven intake after spraying on feed can cause differences in drug intake, and use of the same drug delivery device increases the risk of cross-infection. To resolve these problems, many researchers have developed other dosage forms of ALT. Chen et al. prepared an ALT–hydroxypropyl–β-cyclodextrin inclusion complex aimed at improving the solubility of ALT (15), and to simplify the use of ALT, Wang et al. prepared ALT microcapsules to facilitate mixed feed administration (16), but neither of these authors conducted equivalence studies.

Softgel capsules are active pharmaceutical ingredients or nutrients dissolved, dispersed, or suspended in a liquid or semi-solid filler and encapsulated in a gelatin shell (17). They are a new dosage form developed after tablets and injections. The soft capsule shell is usually composed of gelatin, glycerol, and other components. The shell can prevent oxidation of the capsule contents, and for light-sensitive drugs intended for topical application, titanium dioxide and pigment can be added to further protect the drugs (18, 19). Compared with traditional oral formulations, soft capsules have many advantages in terms of safety, efficacy, and bioavailability; elasticity and plasticity, which can resist moisture and oxidation, thus improving drug stability; and convenience of administration and high patient compliance (20).

In view of the important role played by ALT in sows in regulating the oestrous cycle, influencing uterine development and regulating parturition, and the superiority of the soft capsule formulation. The aim of this study was to screen out the suitable dissolution aid carrier using the shaking bottle method to improve the solubility of ALT, and then encapsulate the content in softgel capsules for *in vitro* dissolution and stability investigation. We carried out a bioequivalence study of the prepared softgel capsules and the commercially available oral solution, to provide a research basis for the clinical application of the softgel capsule of ALT.

## 2 Materials and methods

### 2.1 Materials

ALT (99.7%) was purchased from Xiamen Orijet Biotechnology Co. Ltd. (China). ALT standard with 98% purity and testosterone propionate with 99.8% purity were purchased from China Veterinary Drug Inspection Service (Guangzhou, China). Butyl hydroxyanisole with 99.4% purity and butylated hydroxytoluene with 99.9% purity were purchased from National Institutes for Food and Drug Control (Beijing, China). Soya bean oil (for injection) was purchased from Zhejiang Tianyushan Medicinal Oil Co. Ltd. (Zhejiang, China). Chromatography grade methanol and acetonitrile were purchased from Thermo Fisher Scientific (China). Chromatographic grade formic acid was purchased from Shanghai Aladdin Biochemical Technology (China). Ethyl acetate (EA) and isopropyl myristate were purchased from Guangzhou Chemical Reagent Factory (China). Glycerol triacetate, triacetin, triethyl citrate, and tributyl acetyl citrate were purchased from Shanghai McLean Biochemical Technology Co. Ltd. (China). Gelatin was purchased from Rousselot Gelatine Co. Ltd. (Wenzhou, China). Glycerol was purchased from Nanchang Baiyun Pharmaceutical Co. Ltd. (China). Titanium dioxide and red iron oxide were purchased from Ningbo Yipin Biotechnology Co. Ltd. (China). Other reagents not individually stated were of analytical grade. ALT oral solution (4 mg/ml) was a product of Intervet International limited French factory (France).

### 2.2 Preparation of softgel capsules

#### 2.2.1 Detection of ALT

The concentration of ALT in softgel capsules was determined by HPLC (SIL-20AT; Shimazu, Japan). The wavelength in HPLC was set at 345 nm, an Avantor C18 column [5 μm, 4.6 × 250 mm, Avantor VWR (Shanghai) Co. Ltd.] was used for separation at 35°C, and the mobile phase was acetonitrile: water (98:2, v/v) with a flow rate of 1.0 ml/min. The standard curve of ALT was *y* = 56,065.2*x* – 67,100.1 (*r*^2^ = 0.9996090, *y*: absorption peak area, *x*: concentration of ALT). The absorption peak area had a good linear relationship with the ALT concentration at 5–100 μg/ml, which accorded with detection requirements.

#### 2.2.2 Solubility studies

The shake flask method was applied to determine the equilibrium solubility of ALT in different solubilizers. An excessive amount of ALT was added to a quantitative amount of EA, glyceryl triacetate, triethyl citrate, medium chain triglycerides, and isopropyl acetate to obtain a supersaturated solution. All samples were shaken at 37 ± 0.5°C and 100 rpm for 48 h (THZ-98AB; Shanghai Yiheng Scientific Instrument Co. Ltd., China). After equilibrium, aliquots of 0.5 ml were withdrawn and centrifuged at 10,000 rpm for 10 min. The resultant supernatant was withdrawn, filtered through a 0.22-μm PTFE filter, appropriately diluted. The concentration of ALT in different solvents was determined by HPLC (Section 2.2.1), and three parallels (*n* = 3) were made for each solvent.

#### 2.2.3 Preparation of softgel capsules

The softgels consisted of a capsule shell and contents. Content preparation was based on the results of the solubilizer screening. ALT was weighed and added to the solubilizer and heated with stirring to 45°C. After complete dissolution of ALT, butyl hydroxyanisole and butylated hydroxytoluene were added as antioxidants, and soybean oil (for injection) was added as a solvent to bring the ALT content to 2%. Gelatin, titanium dioxide, and red iron oxide were dissolved in glycerol and distilled water at 78°C. The gelatin was stirred and homogenized for 40 min to obtain the gel solution, which was vacuum degassed and cooled to 50°C. The filling volume of the softgel capsule machine (RJWJ-15; Wuxi Zhongyi Pharmaceutical & Chemical Machinery Co. Ltd.) was set to 1 ml, and the contents were automatically filled and pressed into pills.

### 2.3 Testing of drug contents

ALT concentration was determined in three batches of softgel capsules. One milliliter of the softgel capsule content was measured into a volumetric flask by an internal volume pipette, diluted 500-fold with acetonitrile, and subjected to HPLC to measure ALT concentration (Section 2.2.1).

### 2.4 *In vitro* release study

Dissolution tests were carried out using USP Instrument 2 (paddle) with buffers of different pH (1.2, 4.3, and 6.8) containing 1% SDS as the dissolution medium at 37 ± 0.5°C and 100 rpm. In each test, ALT softgel capsules containing 20 mg ALT were introduced into the dissolution medium at time 0. Samples (2 ml) were collected for analysis at 5, 15, 30, 45, and 60 min and 2 ml dissolution medium at 37°C was supplemented to the dissolution device. The samples were filtered through 0.22-μm membrane filters and subjected to HPLC (Section 2.2.1) to determine the dissolution rate.

### 2.5 Stability studies

To investigate the effect of light and temperature on the stability of ALT soft capsules, capsules were placed in Petri dishes and stored at 40°C and 25°C under a light intensity of 4,500 lx ± 500 lx for 10 days. The concentration of ALT was determined by HPLC on days 0, 5, and 10. Three batches of ALT soft capsules packaged in pharmaceutical grade PP plastic bottles were kept at 25°C/60% relative humidity and 40°C/75% relative humidity for 6 months. The concentration of ALT was analyzed using HPLC (Section 2.2.1).

### 2.6 Bioequivalence studies

#### 2.6.1 Experimental animal

A bioequivalence study was conducted at Guangdong Guanghui Agricultural and Animal Husbandry (Shaoguan, China). Twenty-eight sexually mature gilts (210–220 days old), weighing 112.21 ± 3.69 kg, were selected. Prior to the tests, the gilts were not treated with drugs other than those necessary for immunization and were acclimatized for 7 days in a warm, ventilated environment. Throughout the testing phase, the gilts were fed a full-value ration once daily in the morning and once daily in the evening, with free access to drinking water. All animals were housed and experiments conducted in strict accordance with the norms for the care and use of laboratory animals.

#### 2.6.2 Drug information

The test product was ALT soft capsule (20 mg/ml softgel, ALT concentration), and the reference product was ALT oral solution, Regumate™ (1 ml: 4 mg, ALT concentration), batch No. A766A01, Intervet International limited French factory (France).

#### 2.6.3 Study design

The bioequivalence study was conducted in a single dose, randomized, two-period, two-sequence, cross-over design with a 7-day washout period between treatment. The sexually mature gilts were randomly allocated into two treatment groups: a single dose of 1 ALT soft capsules or 5 ml oral solution (RegumateTM) was administered to the pig. The mature gilts were fasted for 12 h before dosing and fed normally for 2 h after dosing. Five-milliliter blood samples were collected at 0.25, 0.5, 0.75, 1, 2, 3, 4, 6, 8, 12, 24 and 36 h after dosing. Blood samples were quickly transferred to 5 ml blood collection tubes and centrifuged at 4°C for 10 min at 4,000 rpm to separate the plasma. Plasma samples were stored in a refrigerator at −20°C.

#### 2.6.4 HPLC-MS/MS

Blood sample processing was carried out in a dark environment. After natural thawing of plasma samples, 0.5 ml plasma and 0.45 ml acetonitrile containing 50 ng testosterone (internal standard, IS) were added to a 2-ml centrifuge tube, and the solution was vortexed for 10 min, and centrifuged at 14,000 rpm for 5 min. The supernatant liquid was filtered by a 0.22-μm polytetrafluoroethylene filter, and analyzed by LC-MS/MS (Shimadzu, Guangzhou, China) using a CNW Athena C18 column (2.1 × 50 mm, 5 μm; CNW Technologies, China) with a flow rate of 0.3 ml/min and injection volume of 2 μl at 30°C. The mobile phases were 0.1% formic acid solution (A) and acetonitrile (B), and the elution procedure is shown in Table 1. The MS detector was operated with multiple reaction monitoring scan mode at *m*/*z* 311.30 → 227.15 for ALT and m/z 289.20 → 109.10 for IS. The concentration of the standard curve was 1, 2, 5, 10, 20, 50, and 100 ng/ml. The limit of detection and the limit of quantification are the minimum concentrations that result in signal-to-noise ratios of at least 3 and 10, respectively, under the specified analytical condition.

**Table 1:** LC-MS/MS elution procedure.

**Time (min)**	**Flow rate (ml/min)**	**A (%)**	**B (%)**
0.01	0.3	50	50
3.00	90	10
	5.50	90	10
	5.60	50	50
	8.00	50	50

#### 2.6.5 Analysis of pharmacokinetic parameters

The main pharmacokinetic parameters (*t*_1/2_, *C*_max_, *T*_max_, AUC_0−*t*_, and AUC_0−∞_) were calculated using the Phoenix 8.4 non-atrial model with multifactorial ANOVA and bioequivalence was calculated using a two-sided *t*-test and 90% confidence interval.

### 2.7 Statistical analysis

All data are presented as mean ± standard deviation, and *t*-test statistical analysis was conducted using SPSS version 16.0. The statistically significant differences were expressed as *P* < 0.05 and *P* < 0.01.

## 3 Results

### 3.1 Preparation of ALT softgel capsules

**Figure 1:**
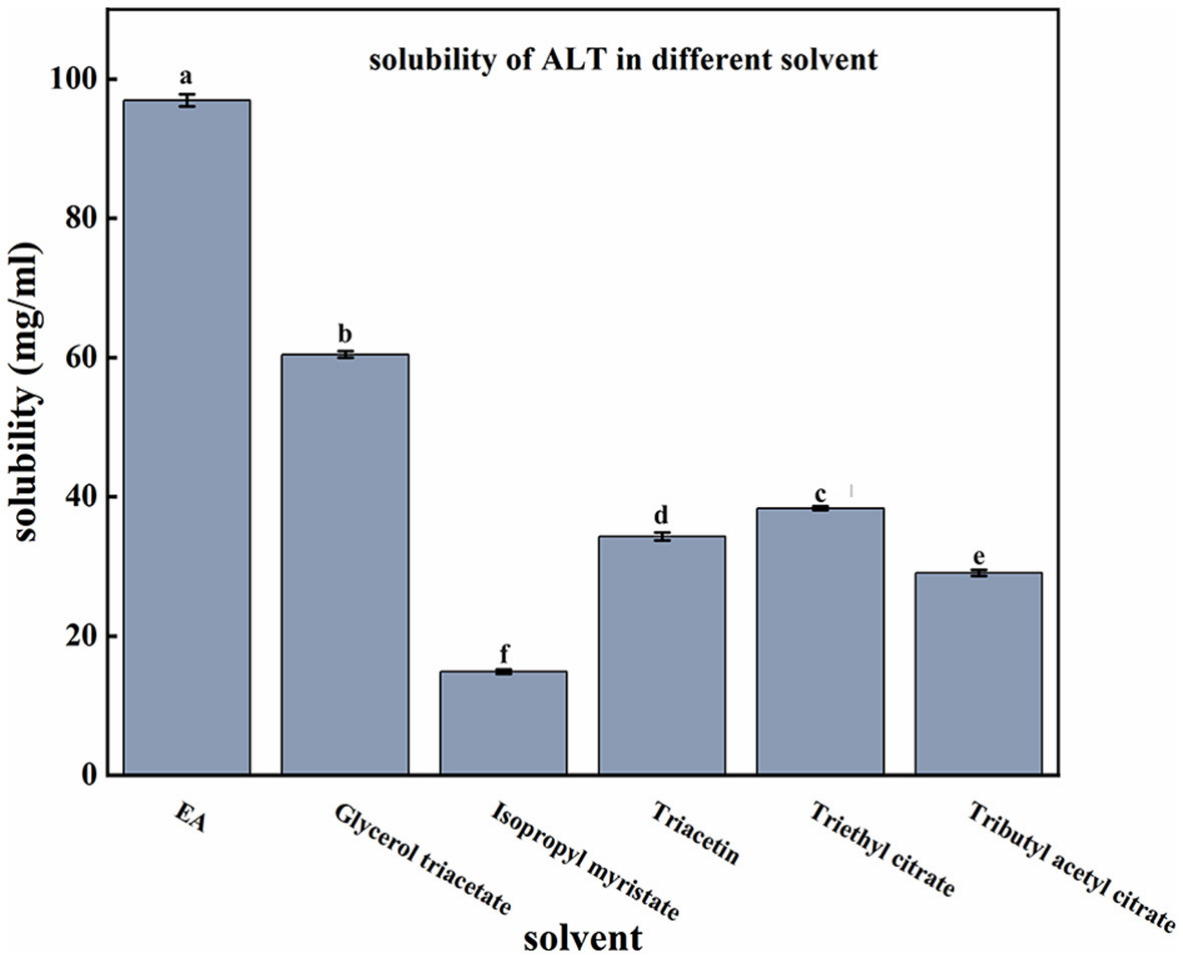
Different solvents on the solubility of ALT. Each value represents the mean ± SD (*n* = 3).

The solubility of ALT in different solvents was: EA > glycerol triacetate > triethyl citrate > triacetin > tributyl acetyl citrate > isopropyl myristate (Figure 1), and the solubility in EA was 96.97 ± 0.88 mg/ml, so EA was chosen as a co-solubilizing carrier to improve the solubility of ALT. Concentration determination showed that the prepared softgel capsules had a specification of 20.09 ± 0.05 mg/capsule (Table 2), which met formulation targets.

**Table 2:** Softgel ALT Concentration determination results.

**Batch**	**Softgel specifications (mg/capsule)**	**Average (mg/capsule)**	**RSD (%)**
1	20.10	20.09 ± 0.05	0.29%
2	20.10		
3	20.20		

### 3.2 *In vitro* release study

**Figure 2:**
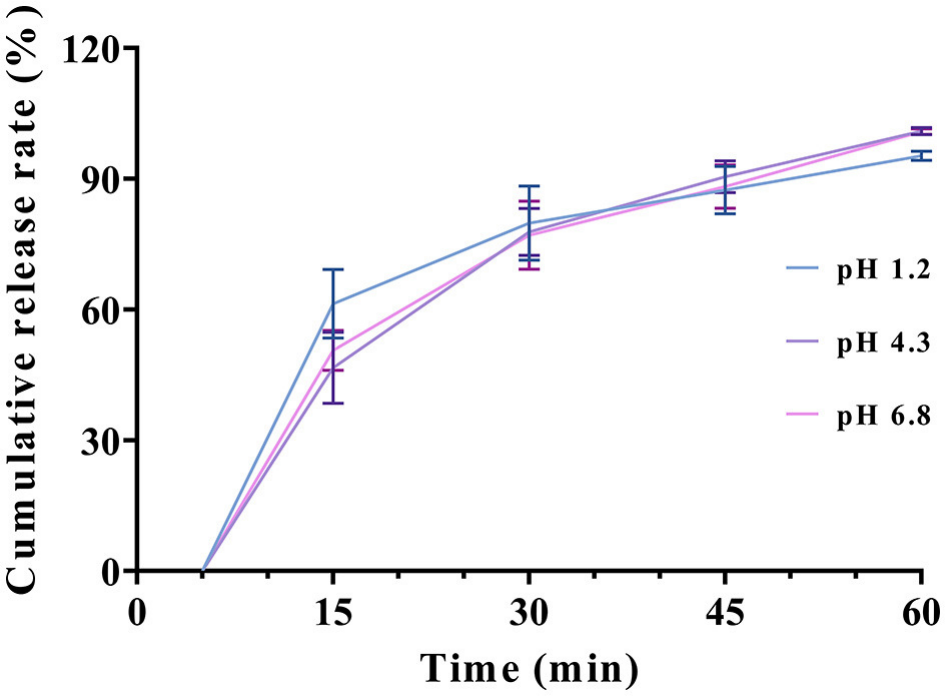
Release curve of ALT soft capsule in different dissolution media.

The ALT softgel capsules were examined for their *in vitro* release properties. Figure 2 shows the dissolution curves of the softgel capsule in buffers of different pH, from which it can be seen that the capsules disintegrated within 5–15 min. After that, the contents rapidly spread in the dissolution medium, and the dissolution degree was more than 90% in 45 min, and release was complete within 1 h. This indicates that the drug has good release characteristics in both gastric and intestinal fluids, which lays the foundation for absorption of the drug.

### 3.3 Stability studies

**Figure 3:**
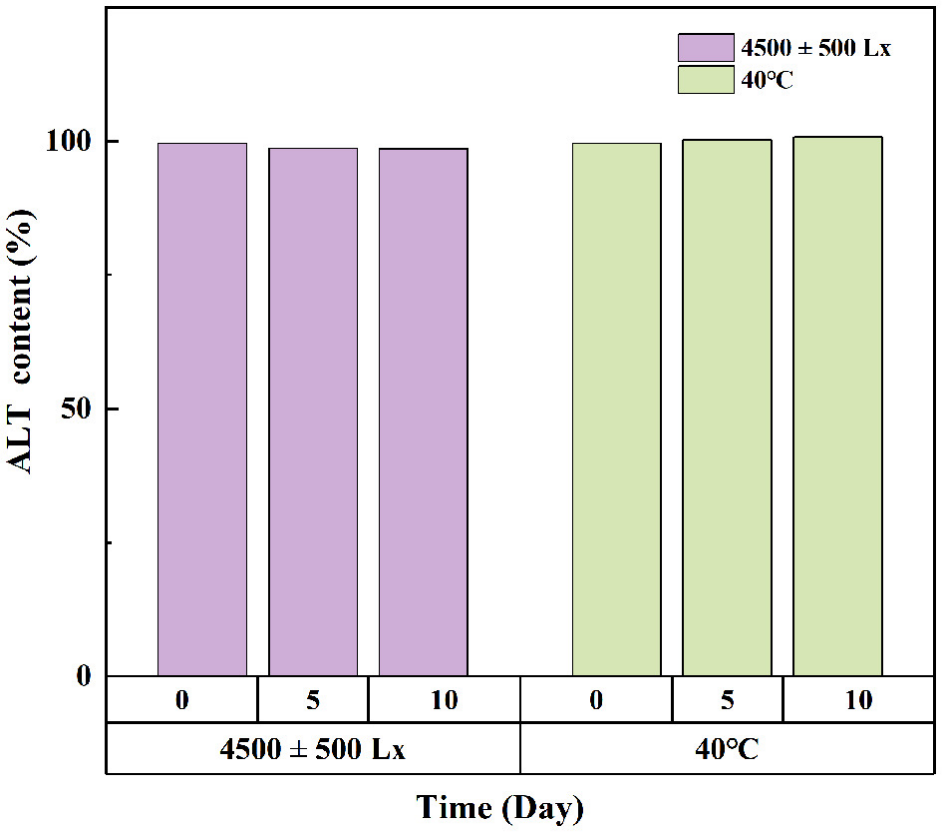
The effects of strong light and high temperature on the storage stability of ALT soft capsule.

**Figure 4:**
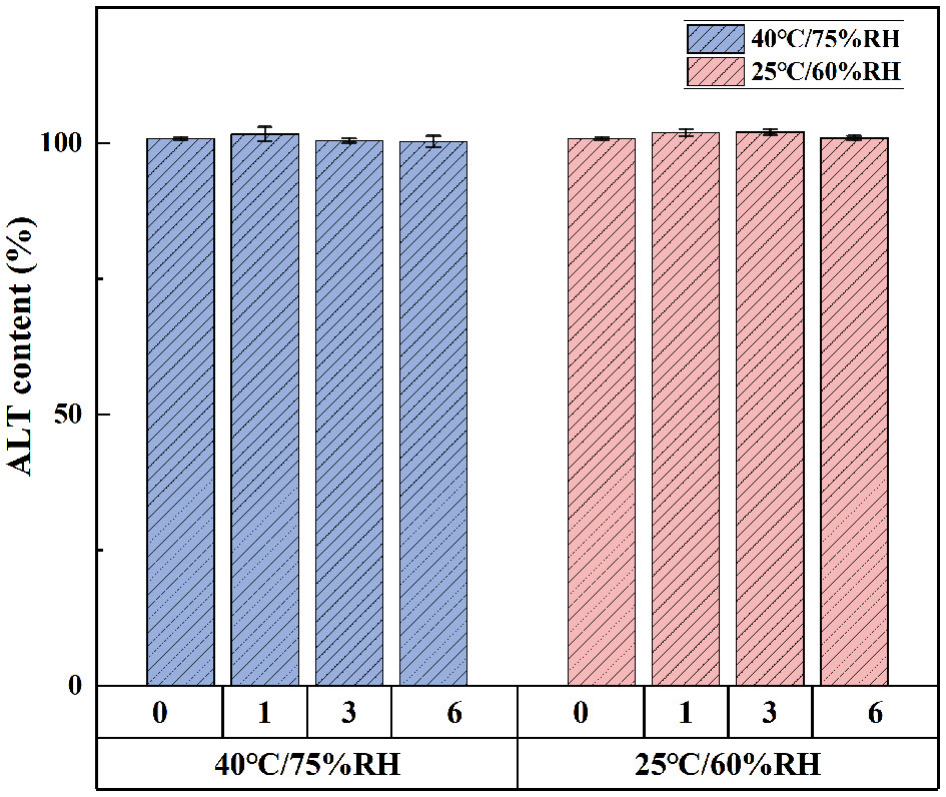
The storage stability of ALT soft capsule at 25°C/60% RH and 40°C/75% RH for 6 months. Each value represents the mean ± SD (*n* = 3).

Under bright light and high temperature of 40°C, there was no significant difference in ALT concentration in softgel capsules over a period of 10 days (Figure 3). The softgel capsules packaged in pharmaceutical grade PP plastic bottles were stored at 25°C/60% relative humidity and 40°C/75% relative humidity for 6 months. The capsules retained >99% of ALT under these storage conditions, suggesting that they were stable under long-term and accelerated conditions for at least 6 months (Figure 4).

### 3.4 Bioequivalence studies

**Figure 5:**
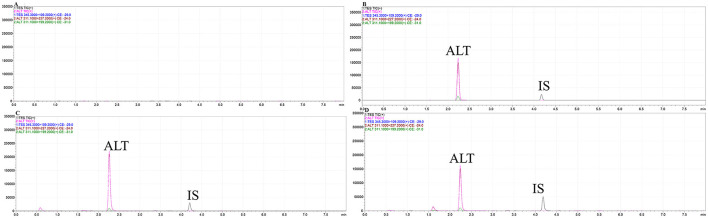
The characteristic ion mass chromatograms of blank gilt plasma **(A)**, blank gilt plasma with addition of ALT standard and IS **(B)**, the plasma from the gilt dosed with ALT soft capsule **(C)** and ALT oral solution **(D)**.

ALT had a retention time of 2.243 min, compared with 4.181 min for the IS. The peaks of ALT and IS showed a good pattern and effective separation, with negligible interference from the endogenous plasma components and good specificity (Figure 5). By adding known concentrations of ALT and testosterone propionate to blank plasma samples, the regression correlation coefficient (*r*^2^) was >0.999 across the concentration range 1–100 ng/ml, demonstrating excellent linearity. Limit of detection was 0.5 ng/ml and limit of quantification was 1 ng/ml. The average extraction recoveries of 2.5, 15, and 80 ng/ml of porcine plasma were 88.79 ± 8.64%, 92.86 ± 5.14%, and 95.94 ± 4.76%, respectively, and 96.20 ± 6.92% for testosterone propionate. Methodology validation results met the assay requirements.

**Figure 6:**
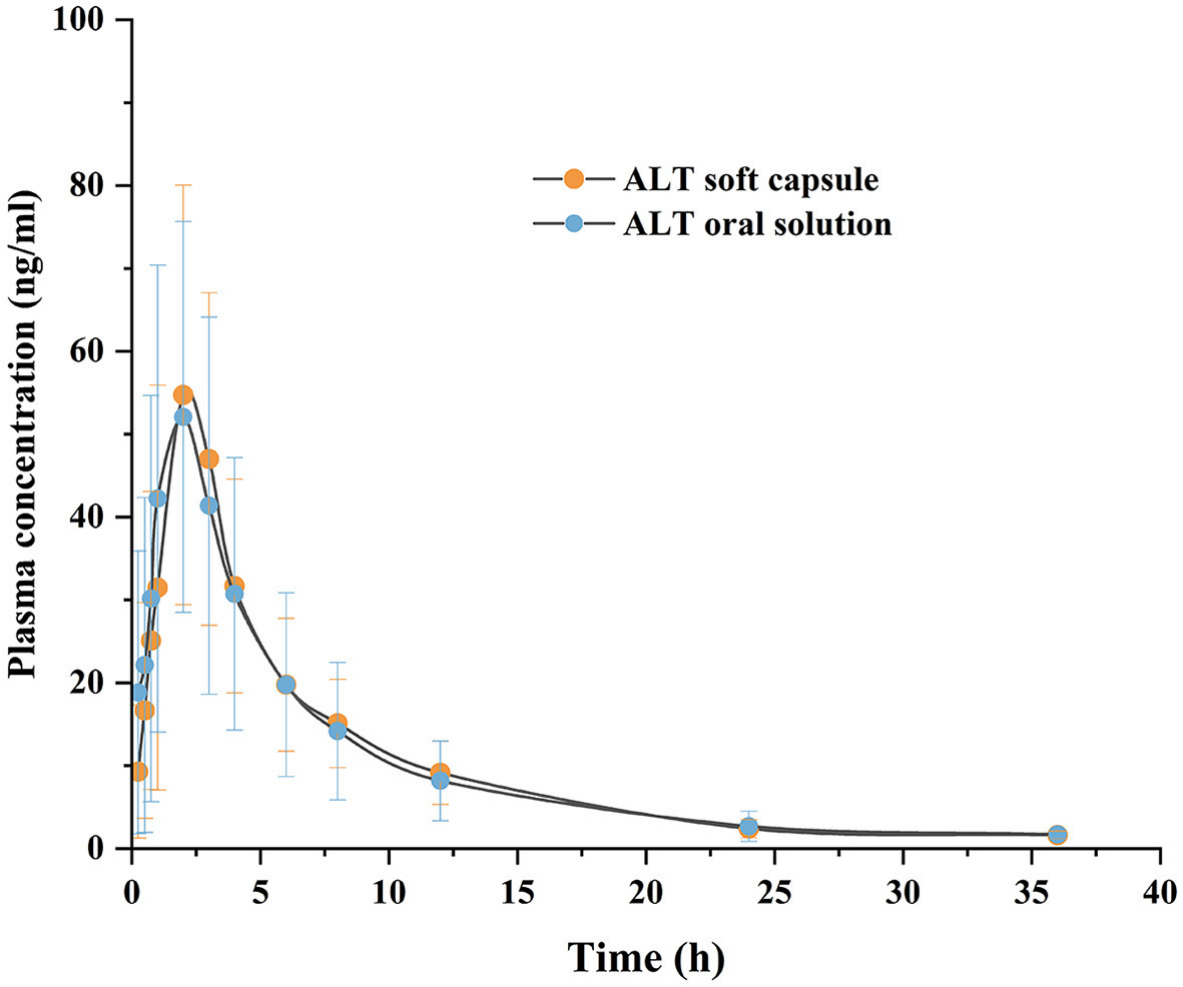
Plasma concentration-time curve following the oral administration of ALT soft capsule and ALT oral solution in gilts. Each value represents the mean ± SD (*n* = 28).

Figure 6 shows the plasma concentration–time profiles of ALT oral administration of softgel capsules and the commercial oral solution at the equivalent dose of 20 mg/pig to gilts. Additionally, Table 3 shows the corresponding pharmacokinetic parameters. *T*_max_ of the softgel capsules was 2.20 ± 0.77 h, *t*_1/2_ was 6.36 ± 1.74 h, and *C*_max_ was 64.65 ± 20.69 ng/ml. The *T*_max_ of the oral solution was 2.19 ± 1.23 h, *t*_1/2_ was 6.57 ± 2.24 h, and *C*_max_ was 67.89 ± 21.92 ng/ml. The results of two-sided *t* tests of the porcine plasma pharmacokinetic parameters obtained after logarithmic transformation are shown in Table 4. The upper and lower limits of the calculated confidence intervals are presented in Table 5. There was no significant difference (*P* > 0.05) in the main pharmacokinetic parameters *C*_max_, AUC_0−*t*_, and AUC_0−∞_ of ALT between the test and reference products after a single oral administration. Both the subject formulation and the reference formulation were shown to be bioequivalent by two-sided *t*-tests and 90% confidence intervals.

**Table 3:** Pharmacokinetic parameters of ALT soft capsule and commercial oral solution.

**Parameters**	**Unit**	**ALT soft capsule**	**ALT oral solution**
*t*_1/2_	h	6.36 ± 1.74	6.57 ± 2.24
*T*_max_	h	2.20 ± 0.77	2.19 ± 1.23
*C*_max_	ng/ml	64.65 ± 20.69	67.89 ± 21.92
*AUC*_0−*t*_	h·ng/ml	361.73 ± 120.57	349.83 ± 146.31
*AUC*_0−∞_	h·ng/ml	379.12 ± 122.06	372.67 ± 150.73

The ALT soft capsule and commercial oral solution were orally administered at the equivalent dose of 20 mg/pig ALT to gilts, respectively. Each value represents the mean ± SD (n = 28). P < 0.05 compared with the commercial oral solution.

**Table 4:** Pharmacokinetic parameters bilateral *t*-test results.

**Parameters**	**t1_ TOST**	**t2_ TOST**	**prob_ 80_00**	**prob_ 125_00**
Ln (*C*_max_)	2.9085	−4.3994	0.0037	0.0000
Ln (AUC_0−*t*_)	5.2472	−3.3304	0.0000	0.0013
Ln (AUC_0−∞_)	4.9313	−3.6294	0.0000	0.0006

**Table 5:** Upper and lower limits of the calculated 90% CI.

**Parameters**	**90% confidence interval**	
	**CI_90_Lower**	**CI_90_Upper**
Ln (*C*_max_)	86.10	106.04
Ln (AUC_0 − t_)	96.19	114.87
Ln (AUC_0−∞_)	94.65	113.07

## 4 Discussion

Softgels have been extensively studied for their ability to improve the bioavailability of poorly soluble drugs and the accuracy of the administered dose. Different types of vegetable oils are commonly used as filling matrices for softgel capsules. In our study, the use of soybean oil as the main solvent for capsule contents was able to avoid the transfer of water from the gelatin shell to the contents and reduce the cross-linking of the gelatin shell and contents, thus improving the stability of the finished softgel capsules (21). Fat-soluble solvents such as EA and isopropyl myristate are often used as the oil phase in the preparation of self-microemulsion to improve drug solubility (22), and our study also used these ester solvents as candidate solubilizing solvents. Solubility studies showed that ALT has maximum solubility in EA, so that was chosen as a co-solubilizing carrier. EA is a short chain, nontoxic, biodegradable ester solvent widely used in food and beverages (23). In the selection of softgel specifications, we successfully prepared ALT softgels with a content specification of 20 mg/capsule based on the clinical single oral dosage, which improved the accuracy of the dosage administered. In addition, the softgel capsule can be administered by mixing in feed, which simplifies the use of the drug and effectively reduces the labor cost.

ALT has low solubility of 0.0166 mg/ml in water (15). To satisfy the leakage condition, we added 1% SDS to the buffer solution, which lowered the interfacial tension between the solution and the surface, and increased the solubility of ALT in the dissolution medium to 1.7126 mg/ml. This satisfied the requirement of 3–5 times the leakage conditions (24). When using the basket method, the capsule shell breaks down into a viscous substance that clogs the mesh of the basket, which in turn affects dissolution of the contents. Pillay et al. encountered the same problem when performing dissolution of softgel capsules (25). Therefore, we selected the paddle method to carry out the dissolution in this study. Disintegration and release of softgel capsules are a prerequisite for drug absorption. By simulating the *in vitro* dissolution of softgel capsules in the gastrointestinal environment, it is possible to predict *in vivo* release and absorption. Our softgel capsules were rapidly dissolved in dissolution media of different pH within 1 h. Compared with the ALT microcapsules prepared by Wang et al. for easy mixed-feeding administration (16), our softgels improved the dissolution and drug loading capacity. The 1-h dissolution rate of HP/β-cyclodextrin/ALT prepared by Wojun Chen et al. was close to 100% (15), which is in agreement with our results, but the present softgel also carried out equivalence studies with the commercially available oral solution.

Temperature, humidity, light, and other storage conditions affect the stability of softgel capsules. Appropriate storage conditions are important to ensure the stability of softgel capsules within the validity period. Under strong light conditions, the concentration of ALT in the softgel capsules was >98%, indicating that addition of titanium dioxide conferred a good shading effect on the capsule shell, which reduced the risk of photodegradation of ALT. Nevertheless, the softgels should be stored as far as possible from light, so as to avoid the denaturation and aggregation of micelles in the capsule shell by strong ultraviolet or visible light irradiation (20, 26). In the 60°C stability test prior to the 40°C elevated temperature test, the capsule shells showed adhesion and leakage on day 5, whereas integrity of the capsule shells was good at 40°C, with ALT levels of 99.74%−100.56%. This indicates that high temperatures during storage significantly increase the risk of cross-linking (27, 28); therefore, softgels need to be stored in a cool place away from light. Other studies have shown that the appropriate storage temperature for softgel capsules is 15–30°C and the relative humidity should not exceed 50%, to ensure the right balance of humidity between the shell and the environment and improve the stability of the softgel capsules (17).

Bioequivalence testing is a method to compare different veterinary products containing the same active substance. In the present study, a bioequivalence test was conducted to compare the pharmacokinetic characteristics and bioequivalence of the prepared softgel capsules with the commercially available ALT oral solution after a single oral administration to hind sows. The differences in *t*_1/2_, *T*_max_, LN *C*_max_, LN AUC_0−*t*_ and LN AUC_0−∞_ between the two groups were not significant by independent samples *t*-test, and the time to peak *T*_max_ was similar. The 90% confidence intervals of the geometric mean ratios of the major pharmacokinetic parameters *C*_max_, AUC_0−*t*_, and AUC_0−∞_ were within the bioequivalence criteria of 80–125%, indicating that the prepared softgels were bioequivalent to the oral solution. In addition, many studies have demonstrated good equivalence between oral solution and soft capsule formulations of the same active ingredient (29–31). Navarro et al. used a two-cycle, two-sequence experimental design to show that cyclosporin A (Cyclavance^®^ oral solution and Atopica^®^ soft capsule) was bioequivalent in Beagle dogs (30). With development of soft capsule preparation technology and further research, administration of solutions filled through the capsule shell is favored by an increasing number of farmers. Our study showed that we have successfully prepared ALT softgel capsules with stable and controllable quality, accurate and convenient dosage delivery, and can be used in the clinic.

## 5 Conclusion

We used the shake flask method to select EA as the solubilizing carrier, and successfully prepared a softgel capsule of ALT with a concentration of 2% and a volume of 1 ml according to the clinical dosage of 20 mg/pig. This improved the accuracy of the dosage and the convenience of using ALT, and reduced the biosafety risk of the farms. The softgels were stable in accelerated and long-term tests at 6 months and bioequivalence studies showed equivalence to commercially available oral solutions, implying that our softgels are ready for clinical use.

## Data Availability

The original contributions presented in the study are included in the article/supplementary material, further inquiries can be directed to the corresponding author.
